# Traditional vs. Contemporary Management Control Practices for Developing Public Health Policies

**DOI:** 10.3390/ijerph13070713

**Published:** 2016-07-14

**Authors:** David Naranjo-Gil, María Jesús Sánchez-Expósito, Laura Gómez-Ruiz

**Affiliations:** Pablo de Olavide University, Carretera de Utrera Km 1, 41013 Sevilla, Spain; mjsanexp@upo.es (M.J.S.-E.); lmgomrui@upo.es (L.G.-R.)

**Keywords:** management control practices, public health policies, enabling and coercive uses of management control

## Abstract

Public health policies must address multiple goals and complex community health needs. Recently, management control practices have emerged to provide a broader type of information for evaluating the effectiveness of healthcare policies, and relate activities and processes to multiple strategic outcomes. This study compares the effect of traditional and contemporary management control practices on the achievement of public health policies. It is also analyzed how two different uses of such practices (enabling vs. coercive) facilitate the achievement of public health policies. Relationships are explored using data collected from managers from public health agencies and public hospitals in Spain. The findings show that contemporary management control practices are more suitable than traditional practices to achieve public health policies. Furthermore, results show that public health policies are better achieved when managers use management control practices in an enabling way rather than in a coercive way.

## 1. Introduction

Well-functioning public health systems are essential for a country’s economic productivity and social development. Public health programs and policies must address multiple goals and challenges, such as preventing epidemics and life-style related diseases or encouraging health behaviors [1]. Health public literature has identified three main general functions of public health systems: (a) assessment of health status and health needs; (b) develop health policy to serve the public interest, and (c) provide necessary health services with quality, equity and accessibility [1,2]. In Spain, like in most Western countries, these functions are developed by public health programs largely based in addressing community health needs, such as programs for: (a) evaluating effectiveness, accessibility, and quality of population-based health services; (b) informing and educating people about health issues and healthy behaviors; (c) diagnosing and investigating health problems and health hazards in the community and (d) developing policies and plans that support community health efforts, among others [3,4]. However, the public health programs do not always get the intended objectives. The increased community pressure and environmental complexity require public health decision-makers to invest in high quality and cost-effective public health initiatives. This scenario demands additional health information processing capabilities, since traditional management control practices are insufficient guide for developing programs with multiple objectives [5,6]. The objective of this paper is to empirically analyze how managers use traditional and contemporary management control practices to manage public health policies.

Traditionally public health management control practices (MCP) focus on collecting and processing data of vital statistics, disease registries, and other surveillance-based resources (e.g., natality, morbidity, mortality and some measure of environmental influences), to provide assistance in planning and operating an agency [7,8]. Traditional MCP include the use of budgeting systems, financial performance measures and reports, and cost-control techniques for decisions. These traditional MCP provide financial accounting performance information, which are unlikely to be sufficient for assessing how different health care activities and processes support a variety of health care policies goals. Financial information and measures are too aggregate and not timely enough to provide appropriate feedback on how agencies are maintaining service quality and timely delivery of their policies. Budget is the traditional practice most used in health public organizations for planning, evaluating and controlling the achievement of objectives and goals. Budgets facilitate organizations to plan their actual operations by encouraging managers to consider problems before they arise. It also helps managers to implement policies by analyzing the variance between intended objectives and the realized goals, which may be used for facilitating efficient resource allocation [8,9]. However, managing health resources effectively requires a much broader scope of information resources to measure the effectiveness and cost of health interventions and policies [10]. As a consequence, public health agencies increasingly adopt contemporary MCP to ensure that managers are provided with a balanced focus on various aspects of health care policies. Balanced information and performance systems link data and measures of customer satisfaction, such as timely and reliable delivery, with other information of key service activities, such as cycle time, which facilitate the adoption of public health programs that simultaneously improve health quality and efficiency [10,11]. Contemporary management control practices include among others, benchmarking, team-based performance measures and balanced information and performance measures. Contemporary practices may support multiple goals by providing comprehensive information, both financial and non-financial, to better control the effectiveness of various management practices in supporting health care priorities [11,12]. The balanced scorecard is one of the contemporary MCP most used in public health agencies, which identifies objectives and measures for four different perspectives: financial, customer satisfaction, internal processes, and innovation and learning [12,13,14]. The balanced scorecard aligns financial measure with non-financial attributes, delineating the holistic notion that financial performance follows operations. It provides an organization’s management with an overarching view of risks and benefits of public health programs. Recent studies illustrate the adoption of the balanced scorecard by a broad range of health care organizations [15,16]. Since not all agencies implement the public health initiatives in the same way, recently several researchers have claimed that more work needs to be done, not only towards understanding the role that MCS play in facilitating the implementation of public health initiatives, but also in understanding how traditional vs contemporary and more sophisticated MCP facilitate governmental authorities to develop public health programs efficiently [17,18].

In the public health sector, all efforts to achieve balanced accountability for health accessibility, equity, cost and equality are more dependent on managers’ attitudes and behaviors than other industries [19,20]. Several researchers have noted that the style of use of management control practices, rather than the simple adoption of them, can affect how public health initiatives and objectives are achieved [21,22]. The management literature has distinguished two main uses of management control practices: coercive and enabling [22,23]. The coercive use refers to more typical top-down management that focuses on central control, focuses on standard setting, evaluating and taking corrective actions [22,23]. Managers adopting a coercive use of MCP view them as a tool that provides diagnoses and information to follow intended goals, but not to open new opportunities. On the contrary, an enabling use of MCP expresses a management style which seeks the participation, focuses on establishing improvement opportunities for internal and external processes, and de-emphasizes target-based control [22,23].

In brief, in this paper firstly we examine the relationship between the achievement of public health policies and the adoption of traditional and contemporary MCP. Secondly we analyze how the enabling vs. coercive use of MCP affects to the efficient of public health programs. The present study extends previous literature by providing empirical evidence about how managers can use traditional and contemporary MCP differently to implement public health policies. Our study shows that an enabling use of contemporary MCP facilitates managers to achieve public health policies with multiple goals. Furthermore, our paper shows that the background of managers affects the different adoption of MCP in organizations. The remainder of this paper is organized as follows. Next section reviews the literature and presents our hypotheses. Then we describe the research method, and we present the results. Finally, the discussion and conclusions of this study are shown.

## 2. Research Hypotheses Development

Nowadays public health organizations rely on MCP to inform managerial decision making and improve operations in areas such as epidemiologic surveillance, health outcomes assessment, program and clinic administration, program evaluation and performance measurement, public health planning, and policy analysis [24,25]. These practices provide real-time information to guide public health decisions. Generally management information and control practices can be defined as a tool that provides different types of information and performance data in the proper time frame to facilitate managerial decision making process and control [25,26]. The importance of health information and control systems has different sources, such as the expanding breadth of data available from multiple public and private sources, the advances in information technology, and the recognition of the power of performance measurement in public health decision making [27,28]. Health care organizations have adopted extensively traditional management control practices, which provide narrow scope information, which is internally focused, mostly financial and historically-based information. Budgets are the most used traditional management control practice adopted for over three decades in health service organizations across different countries [28]. Budgets enable the actual operation of organizations to be measured against the plan or forecast. They have extensively served as a standard for measuring and evaluating financial performance, and as tools to establish the cost constraint for public health programs [28,29].

A renewed examination of public health performance focuses on measuring public health functions in relationship to multiples functions, such as structure, process and outcomes. Structure refers to the cumulative and necessary resources to develop public health processes, such as information, and human or physical resources [29,30]. Processes are the actions through which practitioners seek to identify and address community health problems. Outcomes are the short-term and long-term changes experienced mainly by communities and populations [29,30]. That is, in public health care management, even more than in other industries, the need for a more comprehensive set of information systems, providing financial and operational data, is paramount to manage multiple and interdependent functions. Furthermore, when healthcare programs have to guarantee an additional set of public sector values, such as equity and fairness [31,32]. In this vein, contemporary management control practices, such as balanced scorecard, provide information that is externally focused, non-financial, and future oriented, which facilitate managers to better understand the relationship between healthcare activities, processes and public strategic outcomes.

The balanced scorecard is a sophisticated MCP that provides managers with a broad range of management information, both monetary and non-monetary, in four dimensions (e.g., financial, customer satisfaction, internal processes, and innovation and learning). Even though the balanced scorecard literature is not strongly theory based, from a resource based perspective [33] several authors found that management control systems, such as the balanced scorecard, used in an interactive (diagnostic) way contribute positively (negatively) to the deployment of capabilities of innovativeness and learning. From the balanced use of management control systems emerges dynamic tension which also contributes positively to increase capabilities in a context of high dynamism and uncertainty, such as the healthcare environment [34,35]. The balanced scorecard can be seen as a management control tool that let managers to improve internal and external communications, and monitor how well public health’s structure, resources and activities are aligned with its core functions [36,37]. For example, using infectious diseases as an example, a balanced scorecard could include information about the trends in the incidence of infectious diseases; examination of professional knowledge and supporting for infectious disease programs; analysis of the amount of resources that are allocated to programs and the extent that necessary activities, processes and networks are in place [38,39]. The adoption of a balance scorecard can support the health planning process by informing decision makers how the distribution of resources and services affects population health, and by highlighting health system inequalities. Furthermore it can act as a guide for services by identifying special populations or health care programs with low resources or insufficient capacity to address public demands [40,41].

Despite the increasing trend of adoption of contemporary MCP, such as the balanced scorecard, current evidence from healthcare management literature shows variation on how it is used by managers [23,42]. In this vein, the dynamic environment in which public health agencies operate has triggered a radical shift in the managerial role from a centralized control to a more open and participative control style. With regard to how managers use management information and control systems, we follow the distinction between coercive and enabling use [23,42]. The coercive use of MCP refers to a typical top-down and central control, which focused on comparing planned goals with realized goals, and taking corrective actions for any variance. [42,43]. Managers using MCP coercively focus on providing diagnoses and information to pursue intended goals, but not to consider new opportunities [44,45]. On the contrary, an enabling use of MCP expresses a management style which seeks the interaction and employees’ participation, focuses on establishing new opportunities for internal and external activities and processes. An enabling use is forward-looking and stimulates opportunity-seeking and the emergence of new initiatives [45,46].

**Figure 1:**
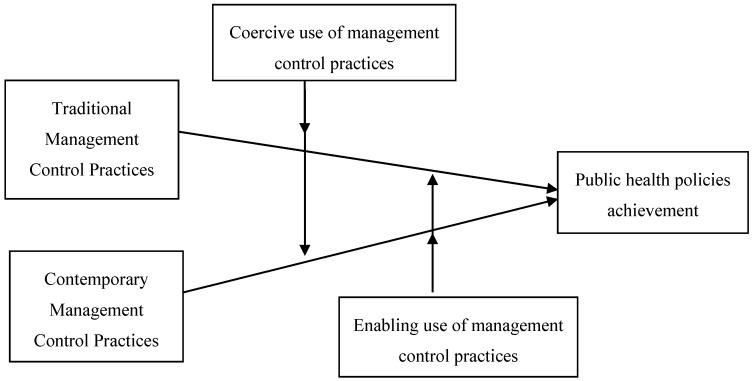
Overall research model.

The achievement of public health policies requires cross-functional interaction and responsiveness to specific community and population health necessity, thus MCP should facilitate interdepartmental planning and coordination more than central performance control. This requires a constant communication and interaction from different departments directed at enhancing workflows, prioritizing activities, and optimizing resource allocation. Contemporary MCP, oppositely to traditional MCP, covers a broad range of information (e.g., financial, operational, future-oriented, internal and external oriented), which would facilitate to achieve public health policies. We expect that an enabling use of contemporary MCP rather than a coercive use of MCP will facilitate the public health policies achievement, since a broad range of information encourage interaction, and stimulate fluent working relationships. In contrast, since traditional MCP focuses on historically-oriented information and expost control, by stressing the measurement and evaluation of unit performance rather than communicating operational decisions, we expect that a coercive use of traditional MCP rather than an enabling use of MCP will facilitate the achievement of public health policies. Figure 1 displays the overall model we examined. This model allows us to analyze how the adoption of traditional vs. contemporary MCP influences the implementation of public health initiatives, and also how the enabling and coercive uses of MCP moderates the impact of public health policies. With these observations in mind, the following hypotheses are formulated:

H1: Contemporary management control practices affect more positively to public health policies achievement than traditional management control practices.

H2: An enabling use of contemporary management control practices has higher impact on the achievement of public health policies than a coercive use of such as practices.

H3: A coercive use of traditional management control practices has higher impact on the achievement of public health policies than an enabling use of such as practices.

## 3. Materials and Methods

Our hypotheses were tested using data from the public health sector in Spain. Under the called new public management paradigm, Spanish governmental authorities encourage managers in public health organizations to achieve public health programs and policies while making annual efficiency saving [46,47]. These reasons assured that the research issues to this study were considered to be relevant for the population. In this paper, we conceptualized the public health system not only as comprising official government public health agencies, but also as comprising other public health-sector organizations, such as hospitals and environmental protection agencies, whose actions also have a significant impact on public health. A questionnaire survey based on standardised and validated items was used to collect information on the adoption and use of management control practices, and the achievement of public health policies. This questionnaire was sent to 218 managers and directors from public health organizations in Spain. A satisfactory response rate was achieved, with 116 useful questionnaires returned (53.22%).

### Measurement of Variables

Managers were asked to indicate the degree of adoption of different management control practices for managing public health policies. Management control practices were classified in traditional and contemporary based on standardized instruments from prior surveys of management control practices, and additional items recommended in recent management accounting literature [48,49]. Managers should indicate in a five point Likert scale from 1 (very low) to 5 (very high) the adoption of traditional control practices, which was measured by three items such as budgeting systems, financial performance reports, and cost-control techniques, and the adoption of contemporary control practices, which was measured by three items such as balanced scorecard, benchmarking and team-based performance techniques. The final scores for traditional control practices and contemporary control practices were measured by the average score of the three items for every practice.

The enabling and coercive uses of MCP were measured using an standardized liker-type instrument from prior accounting literature [32,36]. The instrument was slightly adapted in wording to be understandable in the Spanish healthcare setting based on information from interviews with managers. Respondents were asked to indicate on five-point Likert scale the extent to which they used the MCP for different types of managerial actions. For instance, in the case of coercive use we asked managers to assess the use of MCP for following up preset plans and goals tightly, for managing through the analysis of exceptions and deviation, and for evaluating and control subordinates tightly. In the case of enabling use we asked managers about the use of MCP for encouraging new public health initiatives and policies, for signaling key strategic priorities, and for encouraging the adoption of new actions and internal processes [36,43]. Table 1 shows the exploratory factor analysis revealed that all items loaded higher than 0.50 on the expected constructs: enabling use and coercive use. Cronbach alpha and cumulative variance were also satisfactory [50].

Finally the achievement of public health policies was measured with a standardised instrument that was based on public health care literature and Spanish governmental policy documents [51,52], which are focused on the effectiveness, accessibility, and quality of personal, community and population-based health services. Respondents were asked to indicate in a five point Likert scale from 1 (very low) to 5 (very high) the extent to which a sets of public healthcare policies were implemented in their organizations. For example managers were asked about the implementation of policies on monitoring health status to solve community health problems, policies on health promotion partnership within the community to support healthy living, or policies to assure a competent healthcare workforce by continuing education and life-long learning. Table 2 shows all the items we used for measuring the variable public health policies achievement, and the results of the factor analysis. The exploratory factor analysis revealed that all items loaded higher than 0.50 on one factor, which explained 57.14% of variance. Cronbach alpha was 0.83, which thus exceeded the recommended levels of 0.70 [50].

## 4. Results

Our hypotheses were analyzed using Partial Least Squares (PLS), which allows smaller sample sizes than covariance-based models (e.g., LISREL). The assessment of the measurement model in PLS is comparable with principle components analysis, while the path coefficients in the PLS structural model are interpretable as β-statistics from ordinary least squares regression [53]. We assessed for discriminant validity of our research PLS model by calculating the average variance extracted (AVE) and comparing this with the squared correlations between our variables. Results were satisfactory, which showed AVE’s higher than the squared correlations in all cases.

Table 3 shows the descriptive statistics of our research variables and the demographic characteristics of managers, such as age, tenure, gender and training and education. Regarding this latter, the management literature shows that differences in the training and education of top managers not only explain differences in decision making, but also in the information techniques and processes they choose to apply in their activities and tasks [54,55]. Clinical-oriented and business-oriented managers will focus and use different elements of an information control system. We measured managers’ background with factual questions about managers’ years of educational and functional experience in the professional (clinical) field and the administrative (general management) field [55,56,57].

Table 4 shows the correlations between the variables. We conduct a preliminary data analysis to evaluate the relationship between the demographic characteristics of managers and the adoption and use of traditional and contemporary MCP. As Table 4 depicts, regarding age of managers, we found that old managers are negatively related to adoption of contemporary MCP for health care management. Furthermore we found that older managers are more inclined to a coercive use of MCP. Regarding the tenure of managers, we found that more tenure managers are inclined to adopt contemporary MCP and to an enabling use of them. Regarding manager’s background (clinical vs. business), we found that clinical-oriented managers are positively related to the adoption of contemporary MCP and to an enabling use of MCP. Oppositely, we found that business-oriented managers are positively related to the adoption of traditional MCP and to a coercive use of them. This means that managers with a business background emphasize different management information and control techniques than do managers with a clinical background. Furthermore, the results show that clinical oriented managers use MCP in a more participating and interactive way than managers with a dominant business background. Regarding gender, we analyzed the relationships between managers and MCP in the male sub-sample (*n* = 96) and in the female sub-sample (*n* = 20). We did not find any significant correlations in both subsamples, except for the use of MCP. Regarding the male sub-sample, we found that male managers are positive related to a coercive use of MCP (0.234, *p* < 0.001), and also positive related to an enabling use of MCP (0.198, *p* < 0.05). Thus, results show that male managers use MCP in a coercive way more than in an enabling way. These results are in line with prior studies that argue that most male managers develop managerial styles of command-and-control in organizations [58,59]. In the female subsample, we found that female managers are positive correlated with an enabling use of MCP (0.219, *p* < 0.001), and negative correlated with a coercive use of MCP, however in this case the correlation was not significant (0.171, *p* > 0.10). Thus we can say that female managers are more inclined to use MCP in an enabling way. These results are in line with prior studies that found that women directors are more likely to interact more easily with subordinates at all levels in the organization, since they possess qualities, such as the capacity to establish cooperative relationships and an aptitude for teamwork [58,59]. Overall, the analysis of the demographic enriched our research hypotheses by showing the profile of managers, as a key antecedent variable to understand how MCP is used for healthcare strategic management.

**Figure 2:**
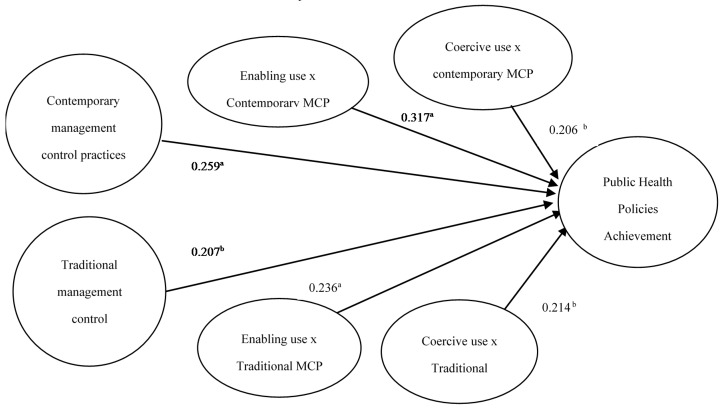
Results from PLS research model. ^a^ Significant at 0.01 level (two tailed); ^b^ Significant at 0.05 level (two tailed).

Figure 2 displays the PLS model tested. Table 5 contains the detailed output statistics of the analysis of the path coefficients in the structural model and reports on the significance of the standardized coefficients that resulted from this analysis, based on a bootstrapping procedure that used 500 samples with replacement. Table 5 shows a positive and significant relationship between public health policies achievement and both the adoption of contemporary and traditional MCP. The path coefficient is higher in the relationship with contemporary MCP than in the relationship with traditional MCP (0.259 vs. 0.207 respectively). Thus, support was found for our hypothesis 1. Results in Table 5 also show a positive and significant relationship between public health policies achievement and the enabling use of contemporary MCP. In this case the path coefficient of the interaction term was 0.317 (*p* < 0.01). The relationship between public health policies and the coercive use of contemporary MCP was also positive and significant, but the path coefficient of the interaction term was 0.206 (*p* < 0.05). Thus, we found support for our second hypothesis. Table 5 shows a positive and significant relationship between public health policies achievement and the coercive use of traditional MCP, where the path coefficient of the interaction term was 0.214 (*p* < 0.05). The relationship between public health policies and the enabling use of traditional MCP was also positive and significant. In this case the path coefficient of the interaction term was higher, 0.236 (*p* < 0.01). Thus, we did not find support for hypothesis 3. Overall, these results may imply that organizations that want to achieve public health policies should encourage managers to use contemporary MCP, such as balance scorecards, rather than traditional MCP, such as budgets. Furthermore they must encourage managers to use MCP in an interactive and participative way rather than in a coercive or restrictive way.

## 5. Discussion 

The objective of this study was to empirically analyze how traditional and contemporary management control practices affect public health policies achievement. We also analyzed two different uses of management control systems (coercive and enabling) and their effect on public health policy achievement. Overall, our results suggest that contemporary MCP act as mechanisms that enable organizations to achieve their public health policies. These findings extend earlier findings by previous studies in the healthcare and management accounting literature [39,55,56]. We found that contemporary MCP, such as the balance scorecard, let integrate multiple data sources available for public health purposes. It facilitates interaction and information flow between different entities feasible. Furthermore, it provides managers with public health information regarding multiple facets, such as preventive services, preventable diseases, and quality of care [49,51]. There are many factors putting pressure on public health policy-makers, such as scarce financial resources, increasingly chronic diseases or rapid technological advances, which highlight the need for comprehensive planning and implementation of healthcare public responses. An effective response requires a systemic approach that encompasses not only action to enhance health care services but also—and as important—measures on disease prevention, healthy lifestyles, and the social determinants of health [3,30,52].

As the healthcare management literature shows, an enabling use of MCP lets managers discuss available management information on critical aspects across organizational levels and functions. Our results seem to confirm the arguments provided by Adler and Borys [23] who argue that the enabling use of control systems supports organization’s balancing multiple strategic objectives. These results also corroborate the findings of previous studies, about the importance of enabling style to promote search of multiple strategic goals and coordination of actions [24,36]. This is in line with the findings in the literature that managers in more successful public healthcare organizations know how to use MCP more effectively [54,55]. Our findings will also help organizations to identify new opportunities and challenges, and to react to them appropriately. In this line, there is a need to empower and train managers in clinical issues to improve the quality, relevance, and understandability of information provide by increasingly sophisticated MCP. Thus, investing in the provision of management information that are understandable to managers with a clinical-orientation and business-orientation, such as through balanced scorecards, which combine financial and operational indicators, may complement such developments [19,27]. 

Our preliminary analyses overall show that managers’ age, tenure, gender and background affects the adoption of traditional and contemporary MCP. Furthermore, these demographics also affect the uses of MCP, which in turn appear to affect the achievement of public health policies. Managers with a dominant clinical background tend to use MCP more in an enabling way than in a coercive way, and seem to emphasize the adoption of contemporary MCP rather than traditional MCP. In contrast, managers with a dominant business background tend to use MCP more coercively than enabling, and tend to adopt traditional MCP rather than contemporary MCPS, such as the balance scorecard. These finding seems consistent with some earlier research in management, for example, those studies that found that managers with a professional and clinical background were more oriented to use control systems in a flexible and adaptive way to manage organizations [19,57]. Furthermore, these findings are also consistent with prior studies that found that managers with an administrative and business background seem to be focused on classical top-down control, with less involvement of subordinates [36,58]. 

## 6. Conclusions

We can conclude that since a majority of indicators in public health reports are derived from only a few data sources, namely vital statistics, hospital discharges, and national or provincial health surveys, the major challenge for public health organizations is to integrate data sources and develop sophisticate management control and information systems that make this information optimally available to public health organizations at all stages of government. Contemporary MCP provides public health care organizations with a greater degree of functional structure coordination that aids in effective public health decision making [51,52].

We can also conclude about the importance of aligning different MCP with different uses in order to avoid frictions that prevent managers from achieving public health policies. Our results show that an enabling use of both contemporary MCP and traditional MCP affects public health implementation positively. Finally, we conclude that the profile of managers affect the use of MCP, such as female managers are more likely to apply a style of using MCP that fosters collaboration, negotiation and democratic participation with the rest of the employees in the organization, which in turn will facilitate the successful implementation of public health care programs and policies [58,59]. In this line, clinical-oriented managers rather than business-oriented managers seem to show behavior that is better aligned with the expected roles of managers for facing dynamic and uncertainty environments, such today’s public health organizations [13,54].

This paper makes several contributions. First, our study has demonstrated that the MCP design can be more or less supportive for achieving public health policies in function of the use of them by managers. Second, our study shows evidence about that an enabling use of contemporary MCP promotes search of new opportunities and coordination, and thus enables public health programs with multiple goals. Third, a practical implication of this paper is that since managers background affects the achievement of public health policies through the use of MCP, managers appointed to implement such policies should be experienced or trained in the use of different types of MCP that provide financial and non-financial information. Finally, this paper has also several limitations, such as lack of ability to test for causal direction and the focus on a single country. Causality cannot be assessed through cross sectional studies like this one. Relying on a single country limited the generalizability of our findings. Clearly, empirical testing of our hypotheses in a different country and using different research methods (e.g., case study or interviews) may provide insight into the external validity of our results.

## Figures and Tables

**Table 1:** Results of factor analysis for the uses of management control practices.

	Factor 1 Coercive Use	Factor 2 Enabling Use
Item 1: following up preset plans and goals tightly	0.782	0.214
Item 2: Managing through the exceptions’ analysis and deviation	0.849	0.186
Item 3: Evaluating and control subordinates tightly	0.772	0.327
Item 4: Encouraging new public health initiatives and policies	0.226	0.834
Item 5: Signaling key strategic priorities	0.274	0.812
Item 6: Encouraging the adoption of new actions and processes	0.183	0.791
Cumulative Variance	32.540%	59.815%
Cronbach Alpha	0.789	0.812

**Table 2:** Results of factor analysis for public health policies achievement.

	Factor 1
Item 1: Monitor health status to identify and solve community health problems	0.722
Item 2: Timely diagnose and identification of health threats in the community	0.760
Item 3: Health promotion partnerships within the community	0.804
Item 4: Develop policies to protect health and guide public health practice	0.751
Item 5: Assure effective entry into a coordinated system of clinical care	0.779
Item 6: Assure a competent healthcare workforce by continuing learning	0.814
Item 7: Monitoring the linkages between public health practice and academic/research (e.g., epidemiological and public health systems studies)	0.785
Cumulative Variance	57.148%
Cronbach Alpha	0.836

**Table 3:** Descriptive statistics.

Variable	Mean	Standard Deviation (SD)	Theoretical Range	Actual Range
1. Adoption of contemporary MCP	2.98	0.32	1.00–5.00	1.00–5.00
2. Adoption of traditional MCP	3.22	0.38	1.00–5.00	1.00–5.00
3. Coercive use of MCP	3.29	0.45	1.00–5.00	1.00–5.00
4. Enabling use of MCP	3.47	0.48	1.00–5.00	1.00–5.00
5. Achievement of Public health initiatives	3.51	0.53	1.00–5.00	1.00–5.00
6. Age of managers	44.6	4.5	–	30–61
7. Tenure	6.7	4.9	–	1–18
8. Clinical-oriented background	2.89	0.71	1.00–5.00	1.00–5.00
9. Business-oriented background	3.07	0.82	1.00–5.00	1.00–5.00
10. Male (Female): 82.75% (17.25%)				

**Table 4:** Correlations among research variables.

	1	2	3	4	5	6	7	8
1. Adoption of contemporary MCP	1.000							
2. Adoption of traditional MCP	0.141	1.000						
3. Coercive use of MCP	0.183	0.211 ^b^	1.000					
4. Enabling use of MCP	0.247 ^a^	0.199 ^b^	0.104	1.000				
5. Achievement of Public health initiatives	0.257 ^a^	0.204 ^b^	0.178	0.195 ^b^	1.000			
6. Age of managers	−0.324 ^a^	0.169	0.231 ^a^	−0.182	0.065	1.000		
7. Tenure	0.219 ^a^	0.152	0.166	0.212 ^b^	0.116	0.224 ^a^	1.000	
8. Clinical-oriented background	0.233 ^a^	0.184	−0.170	0.257 ^a^	0.153	0.126	0.147	1.000
9. Business-oriented background	0.186	0.251 ^a^	0.218 ^b^	0.188	0.161	0.139	0.120	0.074

^a^ Significant at 0.01 level (two tailed); ^b^ Significant at 0.05 level (two tailed).

**Table 5:** Results from PLS analysis (path coefficients).

From:	To: Achievement of Public Health Initiatives
1. Adoption of contemporary MCP	0.259 ^a^
2. Adoption of traditional MCP	0.207 ^b^
3. Enabling use of traditional MCP	0.236 ^a^
4. Enabling use of contemporary MCP	0.317 ^a^
5. Coercive use of traditional MCP	0.214 ^b^
6. Coercive use of contemporary MCP	0.206 ^b^

^a^ Significant at 0.01 level (two tailed); ^b^ Significant at 0.05 level (two tailed).
